# Development of Analytical Methods to Analyze Pesticide Residues

**DOI:** 10.3390/molecules28073074

**Published:** 2023-03-30

**Authors:** Pilar Sandín-España, Thierry Dagnac

**Affiliations:** 1 Unit of Plant Protection Products, Instituto Nacional de Investigación y Tecnología Agraria y Alimentaria (INIA-CSIC), Carretera de La Coruña Km. 7, 28040 Madrid, Spain; 2 Galician Agency for Food Quality—Agronomic and Agrarian Research Centre (AGACAL-CIAM), Unit of Organic Contaminants, Apartado 10, 15080 A Coruña, Spain

## 1. Introduction

Pesticides are compounds applied on crops to eliminate or control pests, diseases and weeds and it is known that their use provides unquestionable benefits in increasing agricultural production. However, pesticide residues can reach the environment and the food chain constituting a potential risk for human health [1]. Therefore, authorities have established high level of protection to human, animal health and the environment, preventing the approval of hazardous substances with a major environmental impact and establishing legal directives to control their levels through e.g., the Maximum Residue Levels. These stringent demands for food and environmental controls make necessary to develop suitable and reliable methods for pesticide residue determinations in environmental samples [2,3]. However, since pesticides and their residues include a variety of compounds with very different physico-chemical characteristics and large differences in polarity, volatility and thermal stability, their determination is usually a challenging task. Furthermore, the low detection levels and the complex nature of the matrices in which the target compounds are present requires efficient sample preparation along with selective and sensitive detection techniques.

The Special Issue “Development of Analytical Methods to Analyze Pesticide Residues” covers eleven contributions (eight original research papers and three reviews). As guest editors, we briefly report an overview of these contributions.

## 2. Results

The sample preparation step which normally includes sample enrichment and clean-up is essential in the development of any analytical method to eliminate matrix interferences prior to detection. Besides, the sample preparation technique is also important to preconcentrate the analytes as they often exist at trace levels prior to detection. This step is of utmost importance for the analytical procedure and it is critical because the determination of the accurate concentration of the pollutants present in the samples depends on its correct implementation. The analytical performance of a solid phase extraction method is influenced by the selection of sorbents that play a vital role in the sensitivity and selectivity of an analytical method.

The review presented by Veloo and Ibrahim [4] provides a general overview on different extraction techniques and sorbents that have been developed in the last 10 years for organophosphorus pesticide determinations in food and water samples, including LLE, SPME, SBSE and SPE using different sorbent materials.

It is known that conventional sample preparation techniques such as LLE and SPE usually involve high consumption of organic solvents, being tedious and time consuming. This has led to the emergence of miniaturized sample preparation methods to overcome the drawbacks of the conventional sample preparation methods [5]. Techniques such as micro matrix solid phase dispersion (µMSPD), ultrasound-assisted extraction (UAE) fabric phase sorptive extraction (FPSE), solid phase microextraction (SPME) and vortex-assisted extraction (VE) among others have received an increasing interest during the last few years.

In this sense, Sergezina et al. [6] developed and validated an UAE method for the determination of 17 fungicides in vine leaf samples using gas chromatography coupled to tandem mass spectrometry to quantify the target fungicides. Furthermore, grape, soil and water samples were analyzed with UAE-GC-MS/MS. Real samples of 17 vine leaf samples were analyzed revealing the presence of 14 of the 17 fungicides at concentrations up to 1000 µg·g^−1^. The concentration levels of the detected fungicides in soils ranged between 0.03 and 15 µg·g^−1^.

In addition, the 14 fungicides were detected in water samples at concentrations up to 100 µg·L^−1^ revealing pesticide transport from leaves to water.

Maragou et al. [7] developed and validated a LC-ESI-MS/MS triple quadrupole method for the determination of 47 pesticides in sludge samples after QuEChERS sample preparation. Pesticides with different physico-chemical properties such as Log Pow values between −0.24 to 7.02 were included. The achieved limits of detection ranged from 0.5 to 50 µg/kg.

The present work studied different chemical classes of pesticides including for the first time the group of pyrethroids in the liquid chromatographic analysis of the complex matrix of sludge. Besides, the method was applied to five sludge samples obtained from an agro-food process in Greece detecting up to 37 active substances.

Since it was first introduced, the QuEChERS procedure has become one of the most widely used techniques for sample preparation in pesticide analysis, mainly in fruits and vegetables. A high number of applications of the QuEChERS method in different matrices has been published for the determination of a wide range of pesticides demonstrating the advantages of QuEChERS in terms of low cost, simplicity, short extraction time and low organic solvent consumption. Furthermore, this approach helps overcome some obstacles derived from the co-extraction of matrix components, such as co-elution, chemical background noise or signal enhancement/suppression.

Hayar et al. [8] developed and validated a method based on QuEChERS and LC-MS/MS for the determination of 33 pesticide residues in vine leaves. Recoveries ranged from 75 to 104%, and repeatability and reproducibility relative standard deviations were less than 20%. The method was applied to the analysis of pesticide residues in 17 market brands of vine leaves processed according to three different preservation methods and sampled from the Lebanese market. In general, the results showed that the dry preserved vine leaves contained the highest levels of pesticide residues compared to brine-preserved and stuffed leaves. Furthermore, the systemic pesticide residues were more frequently found than the contact pesticides. A cocktail of pesticide residues containing up to 13 molecules were detected in the same sample, some at concentrations far exceeding the default MRLs (0.01 mg/kg) set in EU legislation. The systemic fungicides carbendazim, cyproconazole and tebuconazole were more frequently detected than the non-systemic insecticides chlorpyrifos, lambda-cyhalothrin and fenazaquin.

Sandín-España et al. [9] developed and validated a QuEChERS method (citrate-buffered version and PSA with MgSO4 clean-up) followed by LC-ESI-MS/MS for the determination of different class pesticides in persimmon. The method was developed and validated according to EU Commission guidelines and afterwards used for the determination of residues in four field trials. The LOQ achieved for all the compounds was 1.0 µg/kg, except for fludioxonil, for which it was 2.5 µg/kg. Afterwards, after comparing EFSA residue data on apples, as the surrogate major crop, and after conducting a consumer risk assessment, a proposal of residue data extrapolation to set MRLs in persimmon minor crop was performed. The ratio of the MRLs for apple/persimmon varied between 2.5 for boscalid and 1.25 for fluopyram, suggesting that residue extrapolation can be feasible, promoting the process of pesticide registration for minor crops and the settlement of MRL.

Kim et al. [10] developed a single-sample preparation method followed by liquid chromatography–tandem mass spectrometry (LC-MS/MS) for the simultaneous determination of fenpropimorph and fenpropimorph acid in six different livestock products (egg, milk, pork bacon, beefsteak, beef fat and chicken leg meat). Samples were extracted and cleaned using a modified QuEChERS method and the achieved LOQ of 0.005 mg·kg^−1^ fully complied with the CODEX guidelines. The method was successfully applied to real samples obtained from domestic markets.

Rathod et al. [11] validated a method for the determination of 77 multiclass pesticides and their metabolites in *Capsicum* and tomato by gas and liquid chromatography tandem mass spectrometry. The method that involved extraction and purification by a modified QuEChERS approach provided high accuracy, precision, reproducibility and ruggedness, detecting and quantifying pesticide residues below their prescribed EU and FSSAI MRLs.

The concern regarding the presence of pesticides and their residues in environmental waters has increased in the last decades. As a consequence of their widespread usage, a large variety of pesticides have been detected throughout the world in different environmental matrices including surface and ground waters [12]. Therefore, controlling pesticides through monitoring is important to improve, protect and prevent water quality and minimize the potential risks associated to the human consumption.

In this sense, Nakhjavan et al. [13] developed and validated an analytical method for the simultaneous quantitation of 65 pesticides in surface water by liquid chromatography coupled to tandem mass spectroscopy, including one single solid-phase extraction (SPE) procedure. Different parameters that may have an influence on the extraction efficiency were evaluated in this study such as the type or cartridge or elution solvents. All pesticides were recovered at the range of 70–120% at the different spiked levels, and the relative standard deviations (RSDs) were lower than 14%. The feasibility of the method was evaluated on 10 real surface water samples without loss of pesticide recoveries.

Won et al. [14] collate relevant information of stable isotope analyses used under various study fields as an approach for monitoring source tracking. Commonly used analytical methods using liquid and gas chromatography coupled with isotopic ratio mass spectrometry, as well as advanced compound-specific isotope analysis (CSIA) are exposed. CSIA applications are discussed for tracing organic pollutants and understanding chemical reactions (mechanisms) in natural environments. In addition, the authors describe a research direction related to unintended pesticide pollution, which can be adopted and applied in the agricultural field. This study discusses an overview of novel techniques using stable isotope analysis for pesticides and demonstrates the potential of this technology to trace the source and monitor the fate of pesticides in various stages, whilst providing a direction for future research for trace pesticide analysis in the environment and crops.

Another interesting review by Vera-Herrera et al. [15] collects the different methods to assess and/or estimate human exposure to pesticide residues. They can be direct methods by determining the exposure through specific biomarkers in human matrices or indirect methods by estimating the occurrence and subsequent levels in the environment and food matrixes. Extraction techniques reported in environmental matrices analyses were LLE, SPE, PLE, QuEChERS, and Soxhlet. In relation to quantification techniques, the potential of LC–MS/MS and GC–MS/MS is unquestionable. Exposure and risk assessment most widely involved the determination of EDI and hazard quotient (HQ). Therefore, this review included the most recommended techniques to be carried out for each type of study: monitoring of environmental impact due to pesticide pollution in an area under study and relationship with human health, assessment of agricultural practices and human exposure through food consumption, and individual and direct exposure analysis.

## 3. Conclusions

This Special Issue contributes to increasing the knowledge of the recent advances and novel techniques related to the optimization and the validation of analytical methods for the determination of pesticides in different matrices such as food or environmental compartments. The most cutting-edge and widely used techniques for sample preparation in pesticide analysis, including QuEChERS and miniaturized sample preparation methods, are described along with the unquestionable techniques GC-MS/MS and LC-MS/MS for the detection and quantification of pesticides.

## References

[1] El Sherif D.F., Soliman N.H., Alshallash K.S., Ahmed N., Ibrahim M.A.R., Al-Shammery K.A., Al-Khalaf A.A. (2022). The Binary Mixtures fo Lambda-Cyhalothrin, Chlorfenapyr, and Abamectin, against the House Fly Larvae, *Musca domestica* L. Molecules.

[2] Celeiro M., Vazquez L., Nurerk P., Kabir A., Furton K.G., Dagnac T., Llompart M. (2020). Fabric phase sorptive extraction for the determination of 17 multiclass fungicides in environmental water by gas chromatography-tandem mass spectrometry. J. Sep. Sci..

[3] Villaverde J.J., Sevilla-Morán B., López-Goti C., Calvo L., Alonso-Prados J.L., Sandín-España P. (2018). Photolysis of clethodim herbicide and a formulation in aquatic environments: Fate and ecotoxicity assessment of photoproducts by QSAR models. Sci. Total Environ..

[4] Veloo K.V., Ibrahim N.A.S. (2021). Analytical Extraction Methods and Sorbents´ Development for Simultaneous Determination of Organophosphorus Pesticides’ Residues in Food and Water Samples: A Review. Molecules.

[5] Celeiro M., Facorro R., Dagnac T., Llompart M. (2018). Simultaneous determination of trace levels of multiclass fungicides in natural waters by solid-phase microextraction-gas chromatography-tandem mass spectrometry. Anal. Chim. Acta.

[6] Sergazina M., Vazquez L., Llompart M., Dagnac T. (2021). Occurrence of Fungicides in Vineyard and the Surrounding Environment. Molecules.

[7] Maragou N.C., Balayiannis G., Karanasios E., Markellou E., Liapis K. (2021). Targeted Multiresidue Method for the Analysis of Different Classes of Pesticides in Agro-Food Industrial Sludge by Liquid Chromatography Tandem Mass Spectrometry. Molecules.

[8] Hayar S., Zeitoun R., Maestroni B.M. (2021). Validation of a Rapid Multiresidue Method for the Determination of Pesticide Residues in Vine Leaves. Comparison of the Results According to the Different Conservation Methods. Molecules.

[9] Sandín-España P., Mateo-Miranda M., López-Goti C., Seris-Barrallo E., Alonso-Prados J.L. (2022). Analysis of Pesticide Residues by QuEChERS Method and LC-MS/MS for a New Extrapolation of Maximum Residue Levels in Persimmon Minor Crop. Molecules.

[10] Kim S.W., Lim D.J., Kim I.S. (2021). Simultaneous Analysis of Fenpropimorph and Fenpropimorph Acid in Six Different Livestock Productos Using a Single-Sample Preparation Method Followed by Liquid Chromatography-Tandem Mass Spectrometry. Molecules.

[11] Rathod H.N., Mallappa B., Sidramappa P.M., Vennapusa C.S.R., Kamin P., Nidoni U.R., Desai B.R., Rao S.N., Mariappan P. (2021). Determination of 77 Multiclass Pesticide and Their Metabolites in *Capsicum* and Tomato Using GC-MS/MS and LC-MS/MS. Molecules.

[12] Sandín-España P., Sevilla-Morán B. (2012). Pesticide degradation in water. Pesticides: Evaluation of Environmental Pollution.

[13] Won E.J., Yun H.Y., Lee D.H., Shin K.H. (2021). Application of Compound-Specific Isotope Analysis in Environmental Forensic and Strategic Management Avenue for Pesticide Residues. Molecules.

[14] Nakhjavan B., Bland J., Khosravifard M. (2021). Optimization of a Multiresidue Analysis of 65 Pesticides in Surface Water Using Solid-Phase Extraction by LC-MS/MS. Molecules.

[15] Vera-Herrera L., Sadutto D., Picó Y. (2021). Non-occupational Exposure to Pesticides: Experimental Approaches and Analytical Techniques (from 2019). Molecules.

